# What Do Children with Chronic Diseases and Their Parents Think About Pediatricians? A Qualitative Interview Study

**DOI:** 10.1007/s10995-016-1978-0

**Published:** 2016-03-23

**Authors:** Jerzy Konstantynowicz, Ludmiła Marcinowicz, Paweł Abramowicz, Magdalena Abramowicz

**Affiliations:** Department of Pediatrics and Developmental Disorders, Ludwik Zamenhof Children’s Hospital, Medical University of Bialystok, Waszyngtona Street 17, 15-274 Białystok, Poland; Department of Family Medicine and Community Nursing, Medical University of Bialystok, Białystok, Poland

**Keywords:** Communication, Medical jargon, Patient–doctor relationship, Patient satisfaction, Interview

## Abstract

*Objectives* The aim of this study was to determine how pediatric patients and their parents perceive health care during hospital stays, what are their expectations of doctor behaviors, and which components of care do they consider to be the most important. *Methods* A qualitative descriptive study was carried out using the open interview technique. Twenty-six parents and 22 children undergoing hospital treatment participated. *Results* Our analysis identified two major themes: (1) doctor verbal and non-verbal behaviors, which included informing and explaining, conversations on topics other than the illness, tone of voice and other behaviors; and (2) perceived strategies used by doctors. This category included claims of doctors’ intentional use of medical jargon to avoid addressing parental questions directly. Parents admitted that they did not understand medical vocabulary, but they also thought they might understand more of the medical issues if the doctor spoke using terms comprehensible to them. *Conlcusions* Our study shows the importance of interpersonal relationship affecting patient perception of quality of pediatric care. Parents of pediatric patients perceive that doctors behave in ways that deflect parents’ questions and avoid providing them with medical information. Such behaviors include doctors excusing themselves by saying they are busy and using medical jargon. Medical students and doctors should be trained to communicate effectively with patients and their parents and develop skills to convey information in a simple and comprehensible way.

## Significance

*What is already known?* A number of studies demonstrate the importance of interpersonal relationship affecting patient perception of quality of care, however, published data in pediatrics are limited. Difficulties in the doctor-patient relationship, from the patients’ perspective, mainly result from limited time for interacting; failure to consider the patient’s ideas, concerns and expectations. It is known that insufficient information given to the patient, concerning the diagnosis, causes and treatment of the illness, may considerably decrease patient satisfaction of care.

*What this study adds?* This article shows child’s ability to provide valuable perceptions about hospital care. According to the views of children with chronic diseases and their parents, pediatricians tend to use particular behaviors to discourage patients from engaging in discussions with them. Results suggest that doctors commonly demonstrate haste and limit the duration of their visits, show irritation or purposely speak using medical jargon to avoid addressing difficult issues. Training in effective communication with children and their parents should be implemented in the development of education strategies among pediatricians, interns and medical students. Such an approach may alleviate parents’ insecurity and distrust, and has the potential to improve the quality of care of hospitalized children.

## Introduction

Measuring patients’ experience is essential in assessing the quality of health care, as it offers the possibility to determine which aspects of care are important for patients and which aspects need to be improved [1]. In specialized pediatric care, the opinions and satisfaction of both the child and the parents are important [2, 3]. Over 45 years ago, Korsch et al. [4] described the results of a qualitative study of the doctor–patient interaction based on an analysis of 800 tape recordings of patient visits and interviews with parents. The study revealed the importance of the patient’s own ideas about the illness, the family’s expectations of the medical visit, and the need to deal with those concerns. Korsch et al. provided evidence that numerous communication barriers between a pediatrician and a patient’s mother result in maternal dissatisfaction. The barriers were: a notable lack of warmth and friendliness, failure to take into consideration the patient’s fears and expectations of the visit, lack of clear explanations concerning the diagnosis and causes of the illness, and the use of medical jargon [4]. The importance of receiving information and clear communication related to pediatric care is important for both parents and children [5, 6].

The patient–practitioner relationship is a key determinant of quality in health care, and some generally accepted attributes of a good relationship are: personal concern, empathy, respectfulness, willingness to take time, avoidance of condescension, effort to explain, attention to the patient’s preferences, honesty and plain good manners [7]. According to a report from Poland, it is much more difficult to refine the patient–practitioner relationship, shaped as it is by the personal qualities and expressive behaviors of the physician, than it is to improve accessibility to health care services [8]. These personal qualities strongly affect the patient’s perception of care and thus patient satisfaction, and therefore should be investigated. The patient and family are integral members of the health care team and can provide important information for planning and evaluating health care. It is recommended that their opinions be sought for shaping the research agenda concerning pediatric care [9]. Although there are many standardized instruments to measure the quality of health care for ill children, qualitative methods allow for a more comprehensive understanding of the doctor–patient interaction [10]. Furthermore, results of such qualitative studies in pediatrics may potentially improve the quality of health care, and by consequence, patient satisfaction with care [11].

We conducted a qualitative study involving patients and parents to evaluate how hospitalized children and their parents perceive health care during the hospital stay, what are their expectations of doctor behavior, and which components of care do they consider to be the most important for satisfaction of care. Such an approach provides complementary sources of information on interpersonal aspects of health care, and has the potential to improve the quality of health care of children.

## Materials and Methods

### Research Design and Setting

This qualitative descriptive study was carried out with the interview technique reported elsewhere [12, 13]. Sandelowski [12] recommends this kind of study as the method of choice when straight descriptions of phenomena are desired, allowing for “a comprehensive summary of an event in the everyday terms of those events”.

The study was conducted at the Department of Pediatrics and Developmental Disorders of the University Children’s Hospital in Bialystok, a tertiary care unit typical of academic hospital centers in Poland. It was assumed that both chronically ill children and their parents have many experiences with receiving pediatric interdisciplinary care at different institutions. Their experiences may have been associated with a need for multiple visits, consultations and hospitalizations. At the time of the study the children were undergoing conservative management for different medical conditions in the inpatient pediatric unit. The range of chronic diseases included inflammatory illness (recurrent infections), bone metabolic disease, mineral metabolism disorders, autoimmune and rheumatologic conditions (juvenile idiopathic arthritis, reactive arthritis, dermatomyositis).

### Recruitment

The criteria for choice of participants were the following: the child had a chronic illness, at least one previous stay in the hospital and at least 3 days’ stay in this unit, and the child’s general somatic and mental condition, psychomotor or intellectual development being good enough to participate. We used a maximum variation sampling strategy. This approach of purposeful sampling allows researchers to capture and describe the central themes across demographically varied cases [13]. The enrolment continued until saturation of data was achieved [14]. Finally, 22 children aged 10–16 years, undergoing hospital treatment, and 26 parents (22 mothers, 4 fathers) were interviewed. Informed consent was obtained from the participants and the study was approved by the Ethics Committee of the Medical University of Bialystok, Poland.

### Interviews

During the interviews, both parents and children were separately asked open-ended questions allowing for free expression concerning their experiences of receiving pediatric care. The participants were asked the following questions: What was your previous experience of contacts with pediatricians related to your child’s therapy? What is your experience with the current stay in the hospital? What behaviors of the doctors do you like the most? What behaviors of the doctors don’t you like? What is the most important issue in hospital care?

### Data Collection

After obtaining the consent of a parent and/or child, for the children’s convenience and considering the time the parents could devote, all interviews were conducted during the stay at the hospital. The participants were interviewed in a teaching room at the hospital by a trained interviewer (LM), who was not connected in any way to providing health care for the children. The participants’ convenience, anonymity and privacy during the interviews were guaranteed. The interviews were tape-recorded and transcribed verbatim in full in Polish, thereafter the English translations were performed for the purpose of the present study.

### Data Analysis

Qualitative content analysis was used [12]. The authors read the transcripts of the interviews several times and identified certain themes within the children’s and parents’ answers. At the first stage, any interesting or significant points were underlined in the transcripts. At the second stage of the analysis, all the themes were included in a separate file and a list of all the items was made. The third stage of the analysis involved grouping all the significant emerging themes and moving further into the domain of interpretation. Three researchers (JK, LM and MA) were directly involved in the data analysis, and in cases of discrepancy, a consensus was achieved through critical discussion with a fourth researcher (PA). The quotes used in this paper were representative of the views expressed by the participants and were included to exemplify the interesting emerging themes.

## Results

The majority of studied patients were teenage girls, and most of them were receiving treatment for rheumatologic diseases for at least 2 years. The mean age of the parents was 35 years (range 25–58) and their educational status was predominantly higher level (Table 1).

**Table 1:** General characteristics of study participants

Characteristics	Parents (n = 26)	Children (n = 22)
Mean age (range); years	35 (25–58)	13 (10–16)
Gender		
Female	22	14
Male	4	8
Education		
Elementary	2	22
Technical/vocational	6	–
Secondary	6	–
University	12	–
Chronic health condition		
Yes	–	22
No	–	–

Our analysis resulted in two major themes:

1. Doctor verbal and non-verbal behaviors: informing and explaining; conversations on topics other than the illness; tone of voice and non-verbal behaviors.
2. Perceived strategies used by doctors: shortage of time; medical jargon.

### Doctor Verbal and Non-verbal Behaviors

This category included such elements of the patient–doctor relationship as informing and explaining, conversations on topics other than the illness, the tone of voice and other non-verbal behaviors.

#### Informing and Explaining

The need for information concerning the child’s illness, treatment and health dominated in the parents’ statements. This need was often mentioned by the children as well. The participants talked about the need for information in the context of the ways it was provided, namely the language the doctors used, the consequences of a lack of information (parents’ and children’s anxiety), and trust in the doctors. The parents strongly emphasized their expectation that the doctors give information in a simple and understandable way; parents cannot use the information when it is incomprehensible to them.

They [the doctors] do not use the language of an ordinary man. Maybe they told me what it was but I can’t translate it into my language and understand it with my mind. … They [the doctors] are knowledgeable about it, study about it so many years that it’s normal for them. Maybe they think the language they are using is simple. (mother of a 6-year-old son, age 28, higher education)

Failure to inform about the performed procedures is one of the doctors’ behaviors the children did not like and a potential source of anxiety.

When I’m not informed. I mean I’m ill with something and I don’t know anything about it. I’ve got some negative connotations with it, for example I have some exams done and I don’t know anything about them. And suddenly I start to worry why I have them. It’s kind of strange. (girl, age 16)

Insufficient information was also connected with a lack of trust in the doctor.

When contact with the doctor is poor, parents feel insecure and it’s hard to trust the doctor. Trusting doctors is not easy anyway. Because when a doctor is not willing to describe and explain everything, we are more anxious, we think something wrong is going on. (father of a 3-month-old child, age 30, secondary education)

The parents often connected the paucity of information with the doctor’s shortage of time. Insufficient information concerning the child’s illness and the lack of opportunity to make inquiries of the doctor cause parents to search for information elsewhere, e.g. on the Internet or in books, but they are not sure of the knowledge they acquire.

I wish the doctor just had the time for me as a mother. I would like to ask so many things, but I can see there are often more important issues and he’s busy. I wish I had the comfort to inquire about everything, to be calmer. I would like just to sit down and talk. Oh yes. I would need that the most. … I read. I guess. … On the Internet. Some time ago I found some books on the subject, so I have some information. I’ve learned a lot from this book and I have many conclusions. I think so, but I may be wrong. I don’t know. (mother of a 15-year-old child, age 43, vocational education)

#### Conversations on Topics Other than the Illness

Both the children and the parents appreciated the opportunity to talk with the doctor on subjects other than the illness.

The doctors are kind of open toward the patients, interested in the patient, for example they ask me what I like. … I was lying and reading a book, so they asked me what I was reading. … You can talk just like that, normally, not only about medical things. (girl, age 14)

Parents perceive social talk as having a relaxation effect on the tension connected with their child’s illness.

It’s important that the doctors are involved. … They ask about any detail. How are you doing at school? And how is it going at home? … They are interested in everything. The general life of the patient. It’s very nice. They can even ask what the weather is like in Suwalki. It’s also important. It shows he or she is an ordinary human. … Perhaps it will somehow relieve the tension you have. I think this is the normal approach to a human. (mother of a 12-year-old son, age 38, vocational education)

#### Tone of Voice and Non-verbal Behaviors

The tone of voice was a very frequent signal perceived by the parents and children alike. This paralinguistic quality was also often mentioned as an expected doctor behavior.

This is kind of an ideal conduct. When he or she talks to me with a normal voice. … A kind voice, not shouting but a kind, calm voice. This is what I really like in their attitudes. (boy, age 12)

The participants also pointed out keeping eye contact with the doctor, which they associated with the doctor listening to the patient.

I pay attention to whether he keeps eye contact with me. It’s important. Really. Whether he will raise his head over the papers. It’s very important, because then I can feel I’m in the center of attention, that he’s talking to me. Besides, the calm, quiet voice, it’s also important. A raised tone of voice indicates he is nervous and busy. (mother of a 5-year-old son, age 45, higher education)

While talking to doctors and receiving information, parents could also detect the doctor’s nervousness or uncertainty by observing their gestures. They emphasized that the doctor’s behavior affects the parents’ experience.

Even the doctor’s movements, gestures. You can see if he’s nervous or not. Because it’s really, really visible! For example, when one hand stops suddenly, you can see something is wrong. Not always but sometimes I can see a kind of uncertainty on the doctor’s part. … This doctor is calm, balanced, talks quietly without emotions. … It helps improve the parent’s feeling that, after all, everything is okay. (father of a 2.5-month-old son, age 31, higher education)

Another perceived gesture was the facial expression, especially a smile or lack of it.

For example, their facial expression. Whether they are smiling or not. (boy, age 16)

These analyses show that, from both the parents’ and children’s perspective, informing and explaining in a simple and comprehensible manner appear to be essential for useful communication. Moreover, the possibility of engaging in conversation with the physician on different topics beyond the disease/medical issues as well as the doctor’s non-verbal behaviors are extremely important. Notably, insufficient information-sharing by the hospital staff and doctor’s nervousness raise concerns and anxiousness of hospitalized children and often trigger parental mistrust toward the pediatrician.

### Perceived Strategies Used by Doctors

Parents admit that they do not understand medical vocabulary, but they also think they might understand much of the information if the doctor spoke using terminology comprehensible to them. They associate it with the doctor’s limited time to interact with parents.

It’s hard to inform someone who knows nothing about medicine, isn’t it? This is the problem: using words and terms which mean nothing to us. And the Latin expressions sound like magic charms. Basically, we don’t know what they are talking about. So it’s important to transform the information to a level understandable for an average mother or father. … It requires patience and time, first of all, time. (mother of a 5-year-old son, age 45, higher education)

On the other hand, parents perceive some behaviors doctors use to discourage them from taking up a conversation and avoid answering their questions. According to the participants, doctors display haste and irritation, lack time for communicating or even intentionally use not understandable terms or equivocation to avoid addressing difficult issues. Parents interpret these behaviors as the doctor’s reluctance to let them know as much as the doctors do themselves or as preventing them from worrying more than necessary. They also suggest what a doctor should do and how he/she might encourage parents to ask questions.

The thing is to talk, to explain, not to brush me off. I’m not stupid after all, but I’m not going to ask questions when I see that the person doesn’t want to talk to me. What is important is to have the conversation, to talk and to explain. They [doctors] immediately get angry that someone wants to be as informed as they are. And it’s not true, we just want to ask about some details. And they are angry at once: ‘If you know better, go ahead!’ Oh, how I wish there were some discussion, constructive conversation, on equal terms, because we don’t know many things. I would like the doctor to explain a little from the medical point of view. For example, what to do in the future to prevent different situations. … Sometimes he uses very specialist medical terms. Then he leaves and does not explain much. Sometimes it is so, maybe less often now. It would be nice if the doctor were warm, outgoing, if he asked ‘Is there anything else you would like to know?’ To keep the mother or father informed. … Maybe they don’t want someone to be more knowledgeable than they are, to know something more. But people read anyway, because they want to know. (mother of a 5-year-old daughter, age 32, higher education)

Children also perceive the doctor’s shortage of time and talk about it, but at the same time they can find logical explanations for such behaviors.

I mean, it’s obvious the doctors here don’t have too much time, but… I feel that each examination, even a simple one with a stethoscope, like for example today, was very superficial, very short. But maybe it’s because there’s no need of a longer examination. … Even, um, they just walk, drop in the room just for a minute, I undress, there’s the exam and they leave, without asking about anything. (boy, age 13)

Other doctors use medical jargon to stop the conversation and avoid answering parents’ questions, which is unambiguously interpreted by patients.

If the doctor tries to explain something, he or she uses a lot of specialist terms; often the parents have to guess what they mean. They often avoid the answer. Maybe they don’t want to make the patient worry or maybe for another reason. They avoid it by using very specialist words. And they succeed. … Because sometimes they explain clearly what the problem is and the patient or parent (guardian) understands it quickly. And sometimes they avoid it, using some professional jargon. The patient often needs to guess. … I can often understand, because my university education has helped me to comprehend some words, but if I don’t understand, I ask. I often ask but they [doctors] ignore certain subjects. So I’ve come to understand that if doctors use such specialized language, they are trying to avoid telling me something. (father of a 4-month-old child, age 25, higher education)

In summary, parents with higher education usually expect a special partnership and interactive symmetry during the conversation with pediatricians, and they also offer some solutions which may facilitate these relationships e.g., occasion to ask direct questions of the attending doctor. Parents and children are particularly sensitive to certain non-verbal behaviors of physicians connected with hurry, uncertainty and lack of time. Furthermore, the participants perceive that pediatricians limit the duration of their visits, show irritation or purposely speak using medical jargon. Both parents and children mentioned two aspects as being important in hospital care: the doctor’s technical competence in terms of diagnosis and health improvement; and the doctor’s approach to the patient and individual treatment (bedside manner).

## Discussion

Our research shows that being able to engage in a meaningful conversation with a doctor and obtain information concerning the disease and treatment are important attributes of pediatrician–patient relationship and perception of care for both hospitalized children and their parents. Related to these expectations is the problem of medical jargon used, or even overused, by doctors, as was expressed by participants in the present study, and discussed previously in the literature [15–17].

The present study attempted to solicit and combine the opinions and experiences of pediatric patients and parents, as this approach may reflect the true nature of pediatric care specifically in a hospital setting. Some reports have shown that parental involvement in the management of pediatric disorders is regarded to be significant for effective care, e.g. in controlling behavioral problems [18]. Therefore, an increase in parental engagement in the therapeutic process to address children’s problems seems helpful; indeed, parents’ views on the doctor–patient relationship appear to be as important as their child’s perception.

A particularly important result of our study is the perception among pediatric patients and parents that doctors may purposely use medical jargon to avoid having to give detailed explanations and to discourage patients from asking questions. Although the participants rationalized such behaviors as being for the patient’s good or owing to the doctor’s lack of time, patients and parents still intended to be active participants in their relationship with their doctor. They expressed a desire that the doctor talk to them using simple and comprehensible language. It is possible that a cultural context of providing health care plays a role here, since a more paternalistic attitude of the doctor–patient relationship prevails in Poland. European studies show that adult respondents from Poland were significantly less satisfied with doctors’ communication skills than, for example, respondents from Switzerland and the UK [19]. This has been, at least partly, confirmed by the data from our study. Although parents (especially older persons) may sometimes prefer the paternalistic model of care, the need to be treated individually and equally in the relationship with the doctor was strongly emphasized in the present study. Participants expressed dissatisfaction with the care if the doctor did not ask them about their child’s health. Although published data from Poland regarding patient perception of pediatric care are scarce, a questionnaire-based research of children and their parents supports our view by showing that insufficient doctor’s explanation for medical procedures is key determinant of patient dissatisfaction [20].

It is likely that child and parent perceptions of hospital care services are influenced to some extent by demographic or cultural factors; however, irrespective of the health care system this area of pediatric care research remains extremely important. Our findings indicate that patients’ expectations of pediatric care have not substantially changed since one of the pioneering studies was published nearly half a century ago [4], in that patients and parents still refer to both the “expressive” role (i.e., kindness, understanding, emotional support) and the competence of the provider. Other authors’ research also shows that medical care and treatment as well as information about that care and treatment are issues highly ranked by parents in the context of pediatric care [5]. Furthermore, some recent reports using qualitative methods have demonstrated that treatment barriers to receiving family-focused outpatient pediatric services may lead to parental dissatisfaction, distrust and disturbed relationships with health care providers [21]. The data from our study are also potentially useful for informing methods to improve communication between patient/parent and provider and to reduce patient/parent insecurity. As reported elsewhere, opinions of pediatric patients are an important source of information potentially useful for clinic improvement processes [22]. Child’s ability to provide valuable perceptions about care may be implemented in the development of education strategies among pediatricians. In general, the perspectives provided by children and parents are regarded essential components of high-quality clinical decision-making in the hospital setting. Thus, education and training should be provided, in this context, to all students and pediatric residents in order to increase effective use of professional time [9]. An enhanced learning and practice environment in training may result in benefits for future pediatricians regarding their greater professional satisfaction, and also child and family satisfaction [23].

Our study demonstrates that both children and parents are especially sensitive to the doctor’s tone of voice; thus, verbal communication is likely a critical factor in shaping this relationship. Non-verbal behaviors such as eye contact or gestures were less frequently voiced by the participants as being important. One of our previous studies also found tone of voice to be the most frequently perceived feature of family doctors during patient visits [24].

A potential limitation of our study may be that all patients were hospitalized at a single hospital unit; however, of note, the participants also talked about their experiences at other hospitals. The interviews were conducted in the hospital. We considered doing home interviews, as this would probably have been more convenient for the participants; however, this was not feasible due to limited resources. We did not evaluate differences of themes in relationship to disease characteristics; however, this does not detract from our conclusions. Furthermore, we note that the majority of parental participants had a university education, and so their expectations toward pediatricians may have been influenced by that background. Though we attempted to demonstrate opinions and quotes from both male and female parents, the study may over-represent maternal views and perceptions of care because the majority of parental interviewees were mothers.

## Conclusions

This study shows the importance of interpersonal relationship affecting patient perception of quality of pediatric care. Parents of pediatric patients perceive that doctors have developed strategies for avoiding their questions concerning their child’s disease and care. Doctors do this, parents say, by excusing themselves for being busy, and using medical jargon. Doctors may improve their image in the eyes of parents and pediatric patients by providing pertinent and precise information about the illness, medical procedures and treatments, and by engaging in conversations on “general” topics. Medical professionals, including students, interns and doctors, should be trained in effective communication skills and develop the ability to provide information in a simple and comprehensible way.
